# Antisepsine

**Published:** 1890-06-14

**Authors:** 


### ANTISEPSINE.

This is the name given by Professor Cattani to paramonobromacetanelid, one of the new antipyretics, resembling in many respects antipyrin, antifebrine and phenacetine, being also like them a sedative of morbid nerve excitation as well as a reducer of pyrexia.

But Dr. Cattani finds that it is most valuable as an external application to ulcers and non-bleeding wounds. Under its use pain is relieved, granulations assume a healthier character, and finally disappear, while cicatrisation proceeds aseptically. He employs it with the best results in lacerations of the perinæum and in piles in the form of suppositories of cocoa butter with 10-12 per cent. of the drug. In these conditions the healing proceeds without suppuration, notwithstanding the contact of fæces and urine with the surface of the wound. Elsewhere he uses it as a powder. Unlike iodoform it is inodorous, a fact not without its advantages.

